# Dynamical shapes of droplets of cyclodextrin-surfactant solutions

**DOI:** 10.1038/s41598-022-09267-w

**Published:** 2022-03-28

**Authors:** J. Roberto Romero-Arias, Alberto S. Luviano, Miguel Costas, Aurora Hernandez-Machado, Rafael A. Barrio

**Affiliations:** 1 Instituto de Investigaciones en Matemáticas Aplicadas y en Sistemas, Universidad Nacional Autónoma de México, 01000 Mexico City, Mexico; 2 Laboratorio de Bio-fisicoquímica, Departamento de Fisicoquímica, Facultad de Química, Universidad Nacional Autónoma de México, 04510 Mexico City, Mexico; 3 Department of Condensed Matter Physics, University of Barcelona (UB), Barcelona, Spain; 4 Institute of Nanoscience and Nanotechnology (IN2UB), University of Barcelona (UB), Barcelona, Spain; 5 Instituto de Física, Universidad Nacional Autónoma de México, 01000 Mexico City, Mexico

**Keywords:** Physics, Statistical physics, thermodynamics and nonlinear dynamics, Nonlinear phenomena

## Abstract

We present a series of experiments with droplets of aqueous cyclodextrin-surfactant solutions, in which the volume is reduced after the equilibrium spherical shape is reached. The final shape of the drop after this perturbation is found to be dependent on the concentration of inclusion complexes in the bulk of the solution. These inclusion complexes are formed by two cyclodextrin molecules and one surfactat molecule. We propose a model to describe these dynamical processes. Dipole–dipole interactions on the surface of the drop trigger a competition between water surface tension and dipole–dipole interaction energies. The results of the model reproduce the spherical and rod-like shapes found in the experiments.

## Introduction

Shape of vesicles play a critical role in many processes, including coating, cosmetics, drug delivery, therapeutics and others, in which the scale varies from nanometers to micrometers. As an important example of this is that the cellular intake efficiency of drug delivery depends on the shape of the capsule carrying the drug^1^. Encapsulation of drugs in liposomes has proved to be very promising in cancer treatment^2^. Vesicles and annular rings of surfactant-cyclodextrin complexes have been found at the high concentration regime, well above the surfactant critical micelle concentration^3,4^. Furthermore, producing emulsions with two immiscible liquids one can obtain carrier vehicles of the appropriate size^5^. Usually the droplets are stabilized with a surfactant at low concentration, obtaining spherical carriers with low encapsulation efficiency^2^. On the other hand, it has been demonstrated that other shapes, as rods, are more convenient to use in certain cases^1^. Hydrophobins have attracted great attention due to their roles in fungal growth^6,7^ mostly related to surface-chemical properties. They also present high activity to adhere to surfaces. It remains a challenge to understand the role of their rod-like shape to change the surface affinity to surfaces of superhydrophobic properties^8^, their viscoelasticity and other multifunctional properties^9^ for interface engineering, such as hydrophobic drug solubility and delivery biosensoring^10^. In here we found a way of producing non-spherical shapes of hanging drops (the radius of the drops is 1.68 mm, about half the capillary length of water). by taking advantage of the interesting physical properties of drops of aqueous cyclodextrin-surfactant solutions.

In Ref.^11^ we have studied the rheological behavior of a cyclodextrin-surfactant solution in water in the low concentration regime using the hanging drop technique as a function of the concentration. The striking increase of the viscoelastic response of this system and the unexpected variation of the surface tension when varying the composition was demonstrated to be due to the dipole–dipole interactions of the polar molecular complexes that order themselves on the surface of the droplet.

The surface tension and dilatational rheology of the adsorbed material at the liquid/air interface for the mixture ${\text {C}}_{14}{\text {SO}}_4^-$ (S) + $\alpha$-cyclodextrin ($\alpha$-CD) + water with $R = [S]/[\alpha$-CD] at 283.15K were measured by the hanging drop technique using an image drop profile tensiometer. The experimental setup and the employed methodology are described in detail in Ref.^12^. In the experiment one measures the surface tension and dilatational rheology of the adsorbed material at the liquid/air interface at equilibrium, then, the drop is sucked in to a fraction of its volume forming a thin neck, which is concentration dependent, and one waits until the drop acquires its final equilibrium shape.

**Figure 1:**
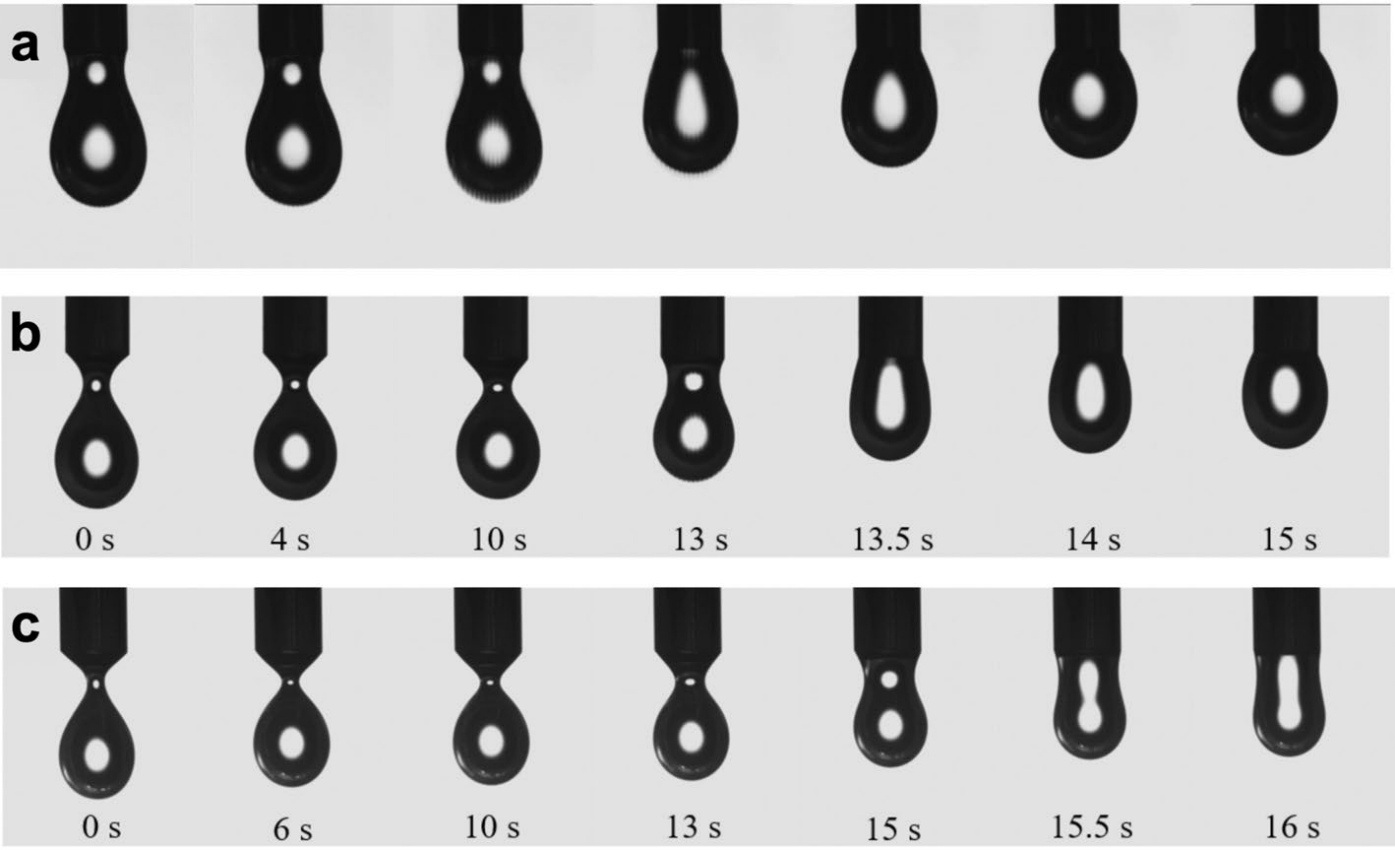
Snapshots of videos (see SM) from the experiments using the mixture *x* mM ${\text {C}}_{14}{\text {SO}}_4^-$ (S) + 10 mM $\alpha$-cyclodextrin ($\alpha$-CD) + water, with $R = [S]/[\alpha$-CD] $= x/10$ at 283.15 K. The snapshots show the drop shape evolution for different ratios. (**a**) $R=0.20$, (**b**) $R=0.34$ and (**c**) $R=0.41$. The dynamic evolution of these shapes is modelled in this work.

In this work we have performed experiments to study the dynamic evolution from an unstable equilibrium state of the droplet shape by changing the concentrations of the cyclodextrin and surfactant in the solution. In Fig. 1, we show snapshots of the dynamic evolution. In a, b and c we show sequences with drops of different *R* values. Initially the drop volume was 20 $\upmu$l and was left to equilibrate (constant surface tension), then the volumen was reduced to 14 $\upmu$l and the drop develops a neck of different widths, depending on R. As time goes by, the snapshots show that the constant volume drop widens its neck, finally forming a sphere in a, or a rod like shape in c and an intermediate shape in b. These snapshots have been extracted form the videos shown in the Supplementary Material (SM). The drop behavior seen in Fig. 1 for different values of the ratio R is remarkable. We observe that the drop changes shape following a favorable curvature to avoid the unfavorable curvature of its neck.

Here we present a mathematical treatment that predicts the dynamical behavior of the experiments shown in Fig. 1, based on a phase field model. Phase-field approaches are suitable to model the dynamics of interfaces that change their shape under certain conditions. For example, in two dimensions, the competition between dipole–dipole interaction between charged molecules and line tension leads to the formation of non-circular domains, like starfish shapes^13–21^. In this work, we propose a dynamical phase-field theoretical model to study the shapes of a drop that initially is taken out of equilibrium. We show that there is a new contribution to the surface energy due to the dipole–dipole interactions because the polar heads of the surfactants, forming the $$(\alpha$$-CD$$)_2$$:S complexes, tend to be on the surface and conserve the total area of the drop, resulting in unusual equilibrium shapes.

## Methods

Our phase field model is defined by means of a total bending energy formalism that can be written as

1 $$\begin{aligned} {\mathcal {F}} = \frac{\bar{\kappa }}{2}\int _{\Gamma } (2H)^{2} ds + \int _{\Gamma }\gamma ds + \int _{\Gamma }\lambda dU_s, \end{aligned}$$

where the first term represents the curvature contribution, the second term is the surface tension energy and the additional third term accounts for the surface energy contributions due to dipole–dipole interactions. Here $\bar{\kappa }$ is the bending modulus, $\gamma$ is the effective surface tension coefficient, and $\lambda$ is a functional of the local surface, since the action of the dipole–dipole energy $U_{s}$ occurs only there.

The dipole–dipole energy density in Eq. (1) is

2 $$\begin{aligned} dU_s = \frac{1}{2}\frac{\mu _e^2 \zeta }{4\pi \varepsilon _0d_*^3 } \frac{h}{d_*^3} ds, \end{aligned}$$

where $$\mu _e$$ is the electrical dipole moment for complexes $$(\alpha$$-CD$$)_2$$:S, $$\zeta$$ is the number of first neighbors, $$\varepsilon _0$$ is the vacuum permitivity, *h* is the thickness of the surface layer where the dipoles are accommodated, $$d_*$$ is an effective distance between dipoles defined as $$d_*=d+0.2c_{_S}^{1/3}$$, where *d* is the mean distance between dipoles used in Ref.^11^, in order to reproduce experimental data^12^ for $${\text {C}}_{14}{\text {SO}}_4^-$$ and *ds* is the element of area.

**Figure 2:**
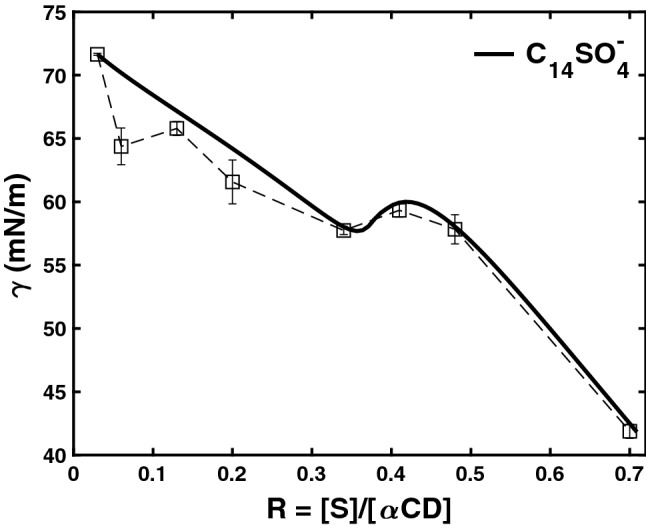
Surface tension of Eq. (3) as function of the ratio *R* for the anionic surfactant at 283.15K (solid line). Square points are experimental values from Ref.^12^ for the mixture *x* mM ${\text {C}}_{14}{\text {SO}}_4^-$ (S) + 10 mM $\alpha$-cyclodextrin ($\alpha$-CD) + water, with $R = [S]/[\alpha$-CD] $= x/10$ at 283.15 K. The dashed line is only to aid visualization. Water surface tension $\gamma _0$ was taken as 72 mN/m. The value of $\eta$ (55 mN m$^2$/mol) was fitted as to match the highest *R* experimental surface tension value. The other parameters where: $\mu _e = 13.96$ D, $\zeta =6$, $h=21.9$ Å  and $d=14.6$ Å^11^.

The behavior of the surface tension as a function of the concentration ratio *R* is shown in Fig. 2. In Ref.^11^ we showed that dipole–dipole interactions are responsible for the effective surface tension behavior as a function of the concentration of complexes in the bulk aqueous solution. These complexes are 2:1 entities i.e. formed by 2 cyclodextrin molecules and 1 surfactant molecule. At the temperature and concentration conditions used in this work we verified, using isothermal titration calorimetry^12,22^, that there is no formation of micelles and/or crystallization in the samples. The surface tension is written as

3 $$\begin{aligned} \gamma = \gamma _0 - \gamma _{{dp}} -\eta c_{S}, \end{aligned}$$

where $\gamma _{{0}}$ is the surface tension of water, $\gamma _{{dp}}$ is the contribution of dipole charges on the surface and the term $\eta c_{S}$ arises from the action of surfactant molecules concentration in the bulk $c_{S}$. The surface tension due to charges is writen as^11^

4 $$\begin{aligned} \gamma _{{dp}}= \frac{\mu _e^2 \zeta }{4\pi \varepsilon _0} \frac{N_{S}}{8\pi d_*^3}\left( \frac{4\pi [S]}{3n}\right) ^{2/3}, \end{aligned}$$

where $N_{S}$ is number of dipoles that could be accommodated on a surface layer and *n* is the number of moles of these dipoles.

In Fig. 2 we show surface tension as function of the ratio *R*, obtained with the model presented in Ref.^11^ and compared it with the experimental findings in Ref.^12^. Notice that for small values of *R* (from 0 to = 0.3) the surface tension decreases reflecting the increasing concentration of surfactant at the liquid/air interface, then there is an intermediate regime in which contribution of the dipoles increases the surface tension and, finally at larger *R* values , where the surfactant becomes dominant, the surface tension of the system dropps sharply. One concludes that the surface tension energy is dominant for small and large *R* and that for intermediate values the bending energy is the one that plays the main role^23^.

A phase-field model of the Cahn–Hilliard type can be defined from Eq. (1), as in Ref.^13^, in which the authors express the free energy $\mathcal {F}$ of the system as an expansion of powers of a smooth scalar field $\phi :\Omega \subset \mathbf {R^3} \longrightarrow \mathbf {R}$, that acts as an order parameter. One of the stable phases, typically defined by $\phi =1$, corresponds to the interior of the volume delimited by the interface located at $\phi =0$, whereas $\phi =-1$ represents the outer environment^13^.

Considering that the element of area in the phase-field approach is

5 $$\begin{aligned} \int _\Gamma ds = \int _\Omega a[\phi ]dV, \end{aligned}$$

where

6 $$\begin{aligned} a[\phi ]= \frac{3|\nabla \phi |^2}{4\sqrt{2}\varepsilon }, \end{aligned}$$

and $\lambda \propto a[\phi ]$ in Eq. (1). Thus, the total free energy in Eq. (1) can be written as

7 $$\begin{aligned} \mathcal {F} = \frac{\kappa }{2} \int _\Omega \left( \mu [\phi ] \right) ^2 dV + \frac{\varepsilon ^2}{2} \int _\Omega \tilde{\gamma }|\nabla \phi |^2 dV + \frac{\varepsilon ^4}{4} \int _\Omega \delta |\nabla \phi |^4 dV, \end{aligned}$$

where

8 $$\begin{aligned} \mu [\phi ] = -\phi + \phi ^3 - \varepsilon ^2 \nabla ^2 \phi , \end{aligned}$$

and $\kappa = 3\sqrt{2}\bar{\kappa }/8\varepsilon ^3$, $\tilde{\gamma } = 6\sqrt{2}\gamma /4\varepsilon ^3$, $\delta = \frac{1}{2}\frac{\mu _e^2 \zeta h}{4\pi \varepsilon _0d_*^6 }(\frac{6\sqrt{2}}{4\varepsilon ^3})^2$. The parameter $\varepsilon$ represents the width of the diffuse interface between the two phases.

The time evolution of the phase-field is set according to the Cahn–Hilliard dynamics, since the volume is supposed to be locally conserved^13–18^. The functional variations of the free energy with respect to $\phi$ must then be subjected to diffusion, yielding a dynamic equation for the phase-field governed by $\frac{\partial \phi }{\partial t} = \nabla ^2 \left( \frac{\delta \mathcal {F}}{ \delta \phi }\right)$^13^, which can expressed in terms of the order parameter and its spatial variations only. The evolution of $\phi$ is described by

9 $$\begin{aligned} \frac{\partial \phi }{\partial t} = \kappa \nabla ^2 \left[ (3\phi ^2-1)\mu -\varepsilon ^2\nabla ^2\mu \right] -{\varepsilon ^2}\nabla ^2 \left\{ \tilde{\gamma }\nabla ^2\phi + \delta \varepsilon ^2\nabla \cdot (\nabla \phi |\nabla \phi |^2) \right\} . \end{aligned}$$

Observe that the two terms in $\{ \}$ compete between them, one is due to the surface tension ($\tilde{\gamma }$) and the other to the dipole interaction ($\delta$). The curvature term ($\kappa$) can induce a rod-like shape, instead of the usual spherical shape, when the slope of the surface tension in Fig. 2 changes sign.

## Results and discussion

By solving Eq. (9), it is possible to find, for instance, the equilibrium shape attained by a drop after a sudden change in volume. Therefore, we performed three dimensional calculations using the same method of integration as in Ref.^13^. We used a finite-difference scheme for the spatial discretization and an Euler method for the temporal derivatives with the appropriate time step of $dt=10^{-4}$ and $\varepsilon =1$, small enough to avoid artifacts^24^.

**Figure 3:**
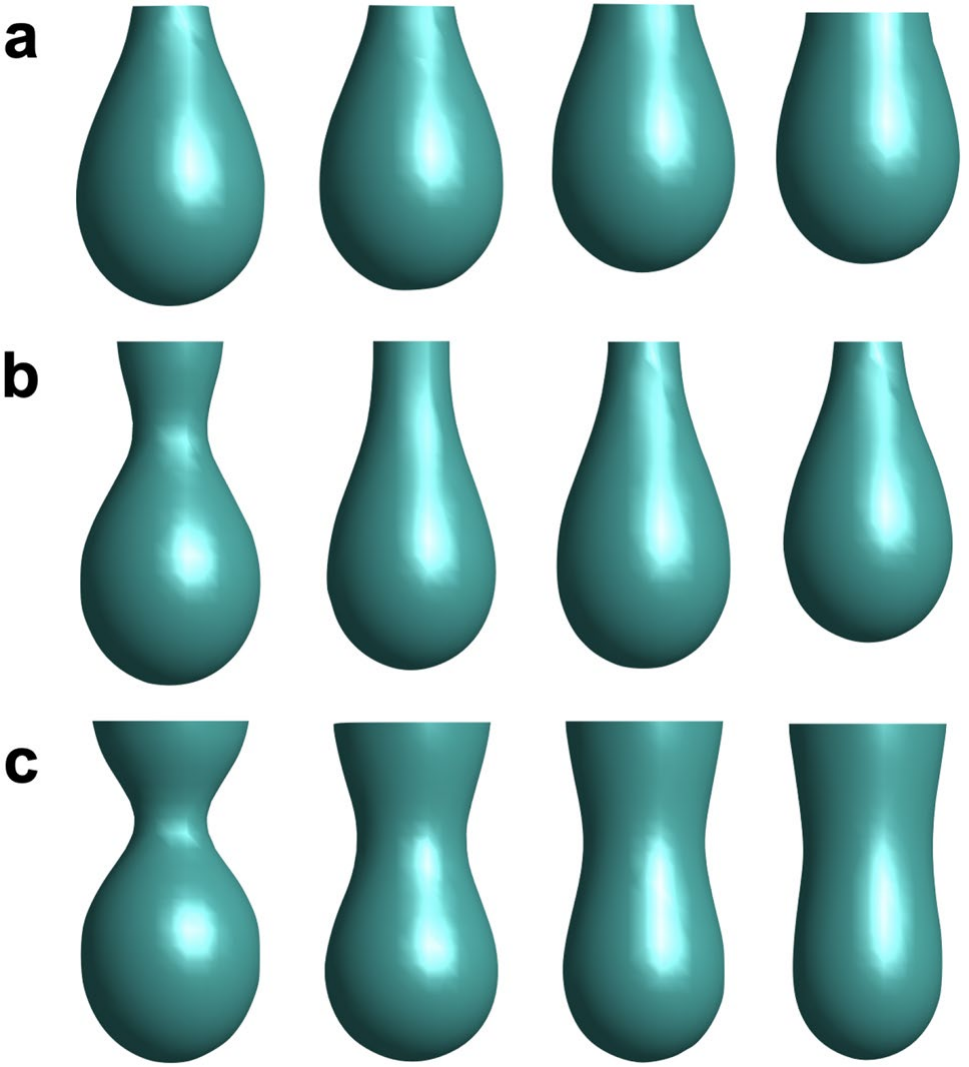
Shapes of the interface $\phi =0$ for different values of *R* obtained form the numerical integration of Eq. (9) . The shapes correspond to $10^5$, $0.5\times 10^6$, $10^6$ and $2\times 10^6$ *dt* interactions. In (**a**) $R=0.20$, (**b**) $R=0.34$ and (**c**) $R=0.41$.

Figure 3 shows the evolution of different drops using the phase-field model by numerically integrating the dynamical Eq. (9). By comparing these shapes with those from the experiment in Fig. 1 we confirm our hypothesis of the competition between surface tension and curvature due to dipole–dipole interactions. When $R=0.20$ water surface tension dominates and the equilibrium shape is spherical, while for $R=0.41$ the curvature energy is important and the drop develops a large thick neck, acquiring the experimentally observed rod-like shape.

As the initial condition we take the droplets that follows the snapshots that corresponds to 10 s in the Fig. 1 for different values of *R*. The numerical integration was performed in a cubic lattice of 40 units. The initial drops have 20 units of height, a radius of 6 units for the spherical part and a neck of radius equal to 2 units. The initial volume is 800 cubic units. We integrated Eq. (9) imposing zero flux boundary conditions on the walls of the domain and $\bar{k}= 10~K_BT$. Taking into account the initial volume of the droplets, these values provide a unit distance of 0.25 mm per pixel and a bending modulus of $k= 5.3~K_BT$. The other parameters take the values: $\tilde{\gamma } =135.4$ mN/m, $\tilde{\gamma } =122.9$ mN/m, $\tilde{\gamma } =127.1$ mN/m and; $\delta =28.9$ mN/m, $\delta =27.7$ mN/m, $\delta = 21.3$ mN/m, for $R=0.20$, $R=0.34$ and $R=0.41$, respectively. In the simulation we get a frame every 2000 steps of size *dt*. Thus, in $2\times 10^6$ *dt* iterations we took 1000 frames. On the other hand, the tensiometer used in the experiment is equipped with a high-resolution CCD camera that saves 123 frames per second^12^. When comparing the number of frames in the simulation with the evolution of the droplets in Fig. 1, we have that the total simulation time is approximately 8 *s*, which is of the same order of magnitude as in the experiment. Under these considerations, the time step *dt* of the simulation is equivalent to $2.5~\upmu$s.

## Conclusions

Here we presented experiments and proposed a dynamical model to describe shape evolution and stationary states of droplets of cyclodextrin-surfactant solutions. We have demonstrated that dipole–dipole interactions play a crucial role in determining the shape of the drops via a competition with surface tension. One can obtain spherical shapes and rod-like shapes by tuning the relative strength of the surface tension and mean curvature. The predictions of the model are in complete agreement with our experimental results. These findings are important, since when using liposomes in drug delivery technologies, the rod like shape is crucial to determine the drug encapsulation control.

The systems that we have studied here contain cyclodextrins that generated a considerable elastic response at interfaces. The essence of this effect is that the surfactant-stabilized cyclodextrin pair generates a dipole and that interactions among dipoles strengthen the surface. This assembly is stiffer than typical surfactants, a property shared by hydrophobins, several of which also show strong elastic surfaces. Interesting filamentous fungi rod-like shapes are observed in hydrophobins and the typical rod-like shape is common in many aerial fungal structures. Hydrophobins are good potential candidates to be studied in the framework of the present theoretical model, and will be the subject of our future research.

## Supplementary Information

Supplementary Information.
